# Efficacy of Repetitive Transcranial Magnetic Stimulation in Fibromyalgia: A Systematic Review and Meta-Analysis of Randomized Controlled Trials

**DOI:** 10.3390/jcm10204669

**Published:** 2021-10-12

**Authors:** Yu-Chi Su, Yao-Hong Guo, Pei-Chun Hsieh, Yu-Ching Lin

**Affiliations:** 1 National Cheng Kung University Hospital, College of Medicine, National Cheng Kung University, Tainan 70428, Taiwan; zac850429@gmail.com; 2 Department of Physical Medicine and Rehabilitation, National Cheng Kung University Hospital, College of Medicine, National Cheng Kung University, Tainan 70428, Taiwan; patchguo@gmail.com (Y.-H.G.); hsieh.pei@msa.hinet.net (P.-C.H.); 3 Department of Physical Medicine and Rehabilitation, College of Medicine, National Cheng Kung University, Tainan 70428, Taiwan

**Keywords:** repetitive transcranial magnetic stimulation, fibromyalgia, meta-analysis, age, dose, parameters, primary motor cortex, dorsolateral prefrontal cortex

## Abstract

This article aimed to investigate the efficacy of repetitive transcranial magnetic stimulation (rTMS) in fibromyalgia. The PubMed, Medline, Cochrane Library, and Web of Science databases were searched for articles published through 14 August 2021. We enrolled only randomized controlled trials. The Cochrane Collaboration risk of bias tool was used for quality assessment. Outcomes were analyzed as standardized mean differences (SMDs) with 95% CIs. The beta coefficient and *p* value were adopted for meta-regression. We included 18 studies comprising 643 participants. A significant reduction in disease influence, as measured by the Fibromyalgia Impact Questionnaire, was observed (SMD, −0.700, 95% CI, −1.173 to −0.228), and the reduction was larger in older patients (*β* = −0.1327, *p* = 0.008). The effect persisted at least two weeks after the final treatment session (SMD, −0.784, 95% CI, −1.136 to −0.432). Reductions in pain, depression, and anxiety were discovered, which persisted for at least two weeks after the last intervention. The effects on pain and depression remained significant up to one and a half months after the final session. No serious adverse events were reported by the included articles. In conclusion, our systematic review and meta-analysis revealed that rTMS is safe and effective for managing multiple domains of fibromyalgia-related symptoms and older patients may have a stronger treatment effect. Larger randomized controlled trials with sufficient male populations are warranted to confirm our findings, detect rare adverse events, and determine the optimal stimulation parameters.

## 1. Introduction

Fibromyalgia syndrome usually presents as widespread pain accompanied by fatigue and psychiatric symptoms [1]. Although pathophysiology of fibromyalgia remains unclear, it is considered to be associated with central nervous system dysfunction causing central sensitization to pain [2]. The prevalence of fibromyalgia in the general population ranges from 0.2% to 6.6% and is more frequent in women [3]. Although fibromyalgia is characterized by widespread pain, it is often accompanied by many other symptoms such as mood disorders, decreased quality of life, impaired work performance, stiffness, fatigue, and physical functioning [3,4]. To capture the total spectrum of the symptoms, the Fibromyalgia Impact Questionnaire (FIQ) was published in 1991, which has been widely used in the assessment of treatment efficacy for fibromyalgia [3,4]. A later Revised Fibromyalgia Impact Questionnaire (FIQR) was published in 2009, which is easier to score but still correlates well with the original FIQ [5]. Due to the wide spectrum of symptoms, multidisciplinary approaches are necessary to achieve optimal management results [6]. This includes both pharmacological and non-pharmacological methods [7,8]. Among them, repetitive transcranial magnetic stimulation (rTMS) acts as a potential choice, with growing numbers of trials performed recently [9].

Randomized controlled trials (RCTs) have reported that rTMS can alleviate fibromyalgia-related symptoms with few adverse events [10,11,12,13,14,15,16,17,18,19,20,21,22,23,24,25,26,27]. Although the mechanism of action of rTMS in fibromyalgia is not fully understood yet, it is believed to modulate the brain areas associated with affective-emotional components of pain, as well as activating the endogenous opioid analgesic system through mediating the motor cortex [28]. No standard protocols have been established so far. Applying both a low-frequency 1 Hz rTMS to the right hemisphere and a high-frequency 10 Hz rTMS to the left hemisphere was found to be effective, and most trials adopted one of the two methods. However, the sample sizes of these experiments were small, and discrepancies existed between the studies. Four articles of meta-analysis [29,30,31] have been published that investigated the efficacy of rTMS in patients with fibromyalgia and detected the sources of between-study heterogeneity [29,30,31,32]. The most recent one, conducted by Sun et al., concluded that rTMS improves pain intensity and FIQ score in patients with fibromyalgia, and the low-frequency rTMS in the dorsolateral prefrontal cortex (DLPFC) region seems to bring an optimal effect regarding the intensity of pain [32]. However, Sun et al. did not measure the modulator effect of different diagnostic criteria for fibromyalgia. Compared with the 1990 version, the 2010, 2011, and 2016 ACR criteria consider additional severity measurements of fibromyalgia-related symptoms and do not include tender point examination [33]. Whether this modification affects the results of rTMS treatment efficacy in patients with fibromyalgia has not been investigated. Besides, considering the wide spectrum of symptoms of fibromyalgia, the modulators for the effectiveness of rTMS measured by FIQ are worth further investigation. Additionally, in patients with major depressive disorder, the effect size of rTMS was determined to be related to age, sex, episode severity, and total rTMS pulses [34,35,36,37]. However, in patients with fibromyalgia, a previous meta-analysis did not recognize a dose–effect response in pain reduction [32]. Whether a dose–effect response measured with FIQ exists is worthy of further research because the FIQ assesses a wider spectrum of symptoms [38]. Furthermore, the influence of age on rTMS efficacy in patients with fibromyalgia has not been surveyed. Finally, although Sun et al. conducted a thorough and comprehensive review of this topic, they did not survey it longitudinally [32]. The duration of the treatment effect of rTMS and its moderators remain unclear.

We conducted a meta-analysis to determine the effect of rTMS for patients with fibromyalgia with longitudinally summarized outcomes. Because fibromyalgia causes a wide spectrum of discomfort and rTMS shows efficacy in multiple categories of symptoms, we chose FIQ/FIQR as the primary outcome. We anticipated filling the knowledge gap of moderator effects of the selected diagnostic criteria, patient demographics, disease severity, and rTMS parameters in the effect size and duration of effectiveness measured by FIQ/FIQR.

## 2. Materials and Methods

This systematic review and meta-analysis was conducted in accordance with the Preferred Reporting Items for Systematic Reviews and Meta-Analyses guidelines [39]. We did not register or publish a prior protocol for this review.

### 2.1. Eligibility Criteria

We enrolled RCTs that reported rTMS treatment effects in patients with fibromyalgia. No limitations were imposed regarding the fibromyalgia diagnostic criteria or rTMS protocol. All retrieved articles were required to include 2 or more treatment arms, one of which must be rTMS and another of which must be a sham or any treatment other than NBS. The publication language was restricted to English.

### 2.2. Search Strategy

We searched the PubMed, Web of Science, Cochrane Central Register of Controlled Trials, and Medline databases. The keywords used were “repetitive transcranial magnetic stimulation” AND “fibromyalgia syndrome.” The search period was from database inception to the present, with the final search conducted on 14 August 2021 (see File S1 for complete search strategy).

### 2.3. Study Selection and Data Extraction

Two reviewers (YCS and YHG) examined titles and abstracts to identify eligible articles. The reference lists of retrieved works were subsequently searched for related papers. When a consensus was not reached between the 2 reviewers, the senior author (YCL) made the final decision. The following data were extracted using a predetermined form: author, publication year, participant characteristics, rTMS details, comparator arm regimens, clinical outcomes, and adverse events. For articles with two or more intervention arms, we divided the control arm equally to form multiple comparisons. We employed the quantile estimation approach proposed by McGrath et al. [40] when medians and interquartile ranges were reported instead of means and standard deviations. National Institutes of Health image software (imagej.nih.gov, accessed on 12 October 2021) was used for outcomes reported as charts [41]. We set the pretest-posttest correlation coefficients to 0.5 if they were unavailable. We contacted the authors as necessary to resolve any uncertainties.

### 2.4. Quality Assessment

We applied the Cochrane Collaboration tool for assessing the risk of bias for quality assessment [42]. The quality of the eligible articles was evaluated by 2 reviewers (YCS and YHG) independently. Reviewer disagreements were resolved through discussion under the supervision of the senior author (YCL). The results were summarized by the Review Manager software version 5.3 (Cochrane, London, UK) and are presented in a graph and summary table.

### 2.5. Statistical Analysis

The primary outcome was FIQ/FIQR score. The secondary outcomes were fibromyalgia-related pain intensity, Brief Pain Inventory (BPI) interference subscale score, McGill Pain Questionnaire (MPQ) score, number of tender points, Beck Depression Inventory (BDI) score, Hamilton Depression Rating Scale (HDRS) score, Hospital Anxiety, and Depression Scale anxiety subscale (HADS-A) score, and fatigue severity scale (FSS) score. The data were extracted for the following time points: at baseline and 2 weeks to 1 month and 1.5 to 3 months after the final rTMS treatment. A meta-analysis was conducted if the outcomes were appropriately reported for 3 or more comparisons in similar populations. We used a random-effects model for effect size pooling; the results are presented as standardized mean differences (SMDs) with a 95% CIs. Between-study heterogeneity was assessed using I^2^, and considerable heterogeneity was defined as an I^2^ of >50% [43]. Subgroup analyses for all outcomes were conducted for the stimulation site, fibromyalgia diagnostic criteria, and frequency of stimulation to identify any moderator effects. A significant difference between effect sizes was indicated by nonoverlapping 95% CIs. Furthermore, to explore the reasons for between-study heterogeneity, we performed post hoc analyses for outcomes with I^2^ values >50%; such analyses comprised random-effects meta-regression exploring the correlations between the effect sizes and the studies’ distinct characteristics. Publication year, age, fibromyalgia disease duration, rTMS frequency, rTMS intensity, pulses per session, total pulses, number of treatment weeks, number of sessions per week, baseline pain intensity, baseline BDI score, and baseline FIQ/FIQR score were treated as quantitative variables. Sex, the fibromyalgia diagnostic criteria, stimulated hemisphere, and targeted brain area were treated as categorical variables. The meta-regression results were considered statistically significant when *p* < 0.05. Funnel plots and Egger tests were used to detect publication bias, and a two-tailed *p* < 0.1 was regarded as statistically significant [44]. We conducted a sensitivity analysis for the primary outcome by removing one trial at a time and analyzing the remaining trials to estimate each study’s contribution to the overall effect size. All analyses were performed in Comprehensive Meta-Analysis software version 3 (Biostat, Englewood, NJ, USA).

### 2.6. Certainty of Evidence

We used the Grading of Recommendations Assessment, Development and Evaluation (GRADE) methodology to assess the certainty of the evidence of the primary outcome. Because our study included only RCTs, the results begin as high certainty, and the final rating depends on the overall risk of bias, imprecision, inconsistency, indirectness, and publication bias [45].

## 3. Results

### 3.1. Study Selection and Description

**Figure 1:**
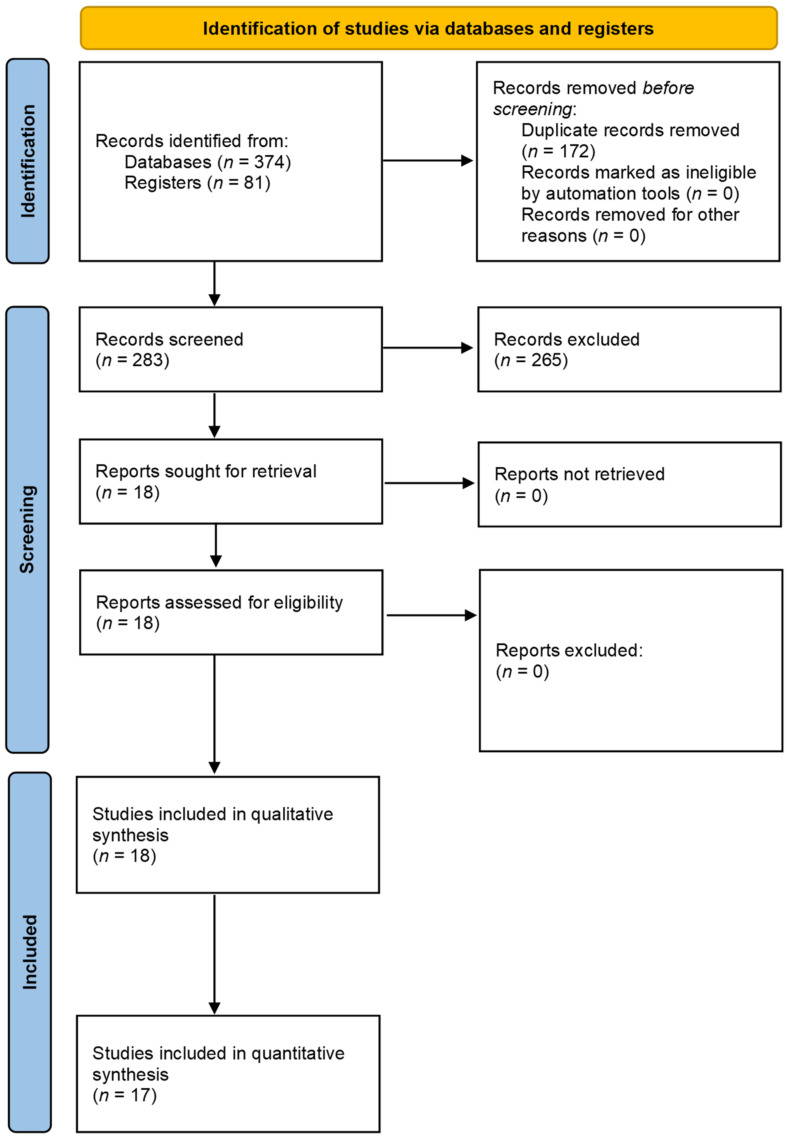
Literature screening process and results.

The initial search returned 455 articles. Eighteen RCTs [10,11,12,13,14,15,16,17,18,19,20,21,22,23,24,25,26,27] with 643 participants entered qualitative synthesis (Figure 1). Two studies [11,18] included two intervention arms; therefore, the control groups for this research were divided for separate comparisons, creating 20 comparisons in total. 17 RCTs entered quantitative analysis for all the outcomes, and one was not included since none of the outcomes reported in the study were in our interest of quantitative synthesis. The included trials’ characteristics are presented in Table 1.

The number of participants ranged from 15 to 86, and the mean age ranged from 40.4 to 53.9 years. Three studies [12,13,17] recruited fibromyalgia patients diagnosed with the 2016 ACR criteria. One article enrolled participants with fibromyalgia diagnosed with the 2011 ACR criteria [18]. Four [10,14,23,24] studies included individuals with fibromyalgia diagnosed with the 2010 ACR criteria. Nine papers [11,15,19,20,21,22,25,26,27] enrolled patients with fibromyalgia diagnosed with the 1990 ACR criteria. One paper [16] did not mention the diagnostic criteria. The number of treatment sessions ranged from 8 to 20. The duration from the first to the last treatment session ranged from 2 to 21 weeks. The total number of pulses ranged from 12,000 to 60,000, and the pulses per session ranged from 1200 to 4000. The targeted brain area was M1 in 10 interventions and DLPFC in nine interventions; one trial [22] did not specify the targeted brain area. Additional data are presented in Table 2.

### 3.2. Risk of Bias Assessment

**Figure 2:**
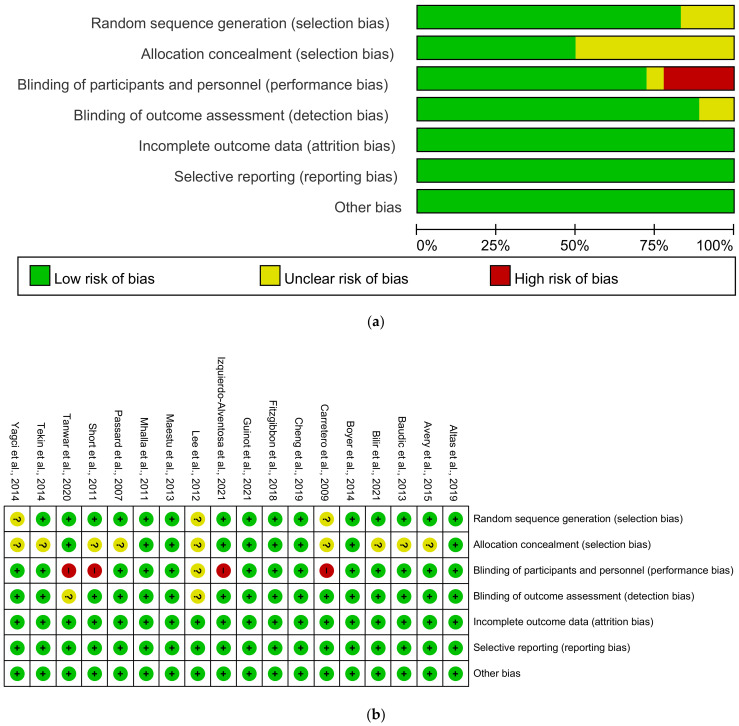
Results of risk of bias assessment. (**a**) Risk of bias graph; (**b**) Risk of bias summary.

Three of the articles [11,19,21] did not report a method for random sequence generation (Figure 2). Nine articles [11,13,15,16,19,21,25,26,27] did not state whether the allocation was concealed. The participants or research team were not blinded in four investigations [12,21,23,25], and blinding was not mentioned in detail in one report [11]. In two studies, outcome assessment blinding was not explained [11,23].

### 3.3. Outcomes

#### 3.3.1. FIQ/FIQR

**Figure 3:**
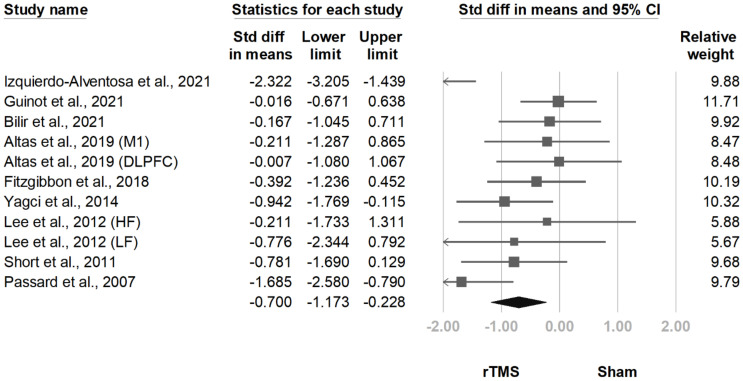
Forest plot of standardized mean differences in Fibromyalgia Impact Questionnaire after treatment. Squares indicate effect sizes of individual studies, lines indicate 95% CI, and diamond indicates the summarized effect size.

**Figure 4:**
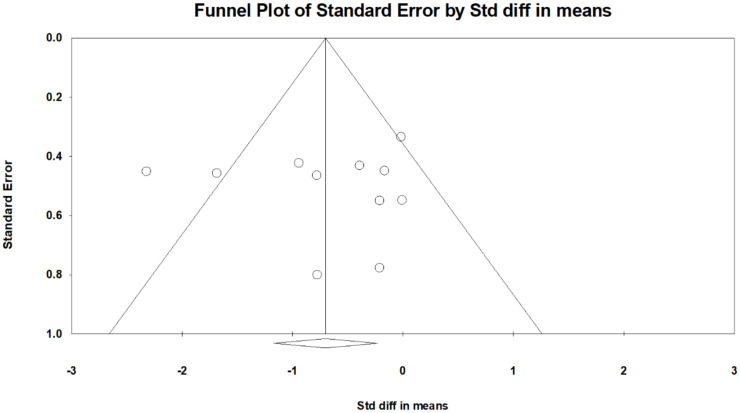
Funnel plot of standardized mean differences in Fibromyalgia Impact Questionnaire after treatment. Each dot indicates a single study, and the diamond indicates the summarized effect size.

The FIQ/FIQR score was mentioned for 13 comparisons derived from 11 articles [11,12,13,14,15,17,18,19,20,24,25]. For 11 comparisons, the FIQ/FIQR scores reported after treatment were significantly lower in the rTMS group (SMD, −0.700, 95% CI, −1.173 to −0.228, I^2^ = 62.8%; Figure 3). The funnel plot (Figure 4) and Egger test demonstrated no publication bias (*p* = 0.91). Sensitivity analysis did not change the results; SMD ranged from −0.518 (95% CI, −0.870 to −0.166), with the study by Izquierdo-Alventosa et al. [12] excluded, to −0.792 (95% CI, −1.286 to −0.299), with the trial by Guinot et al. [14] excluded. The post hoc analyses indicated a larger decrease of FIQ/FIQR scores in trials with older patients (*β* = −0.1327, *p* = 0.008; File S2). No correlations existed with the publication year (*p* = 0.33), sex (*p* = 0.63), disease duration (*p* = 0.85), diagnostic criteria (*p* = 0.44), baseline BDI score (*p* = 0.77), baseline pain intensity (*p* = 0.13), baseline FIQ/FIQR score (*p* = 0.22), stimulation hemisphere (*p* = 0.79), stimulated brain area (*p* = 0.35), rTMS frequency (*p* = 0.75), rTMS intensity (*p* = 0.58), total pulses (*p = 0*.89), pulses per session (*p* = 0.28), number of weeks of treatment (*p* = 0.10), or number of sessions per week (*p* = 0.13).

**Figure 5:**
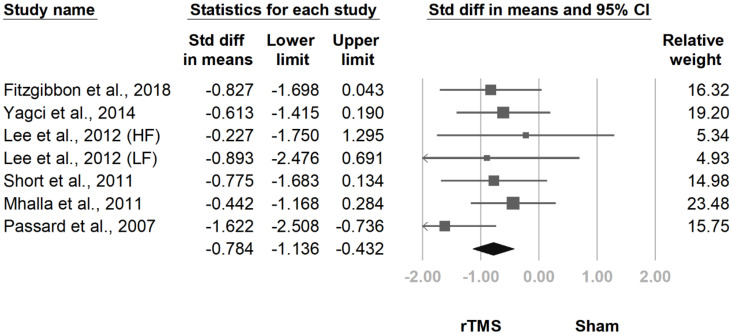
Forest plot of standardized mean differences in Fibromyalgia Impact Questionnaire at follow-up 2 weeks to 1 month after last treatment session. Squares indicate effect sizes of individual studies, lines indicate 95% CI, and diamond indicates the summarized effect size.

At the follow-up at two weeks to one month after the final treatment session, the pooled effect size remained significant (SMD, −0.784, 95% CI, −1.136 to −0.432, I^2^ = 0.0%; Figure 5). The funnel plot and Egger test revealed no publication bias (*p* = 0.99).

#### 3.3.2. Pain Intensity

**Figure 6:**
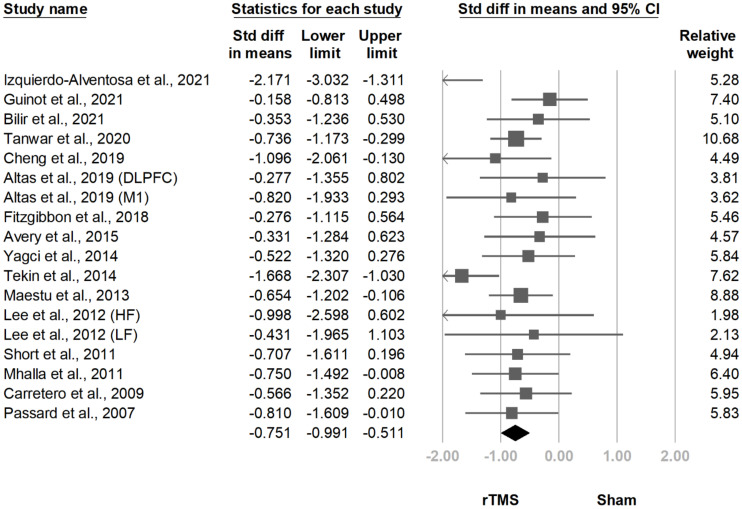
Forest plot of standardized mean differences in intensity of pain after treatment. Squares indicate effect sizes of individual studies, lines indicate 95% CI, and diamond indicates the summarized effect size.

Eighteen comparisons derived from 16 investigations [10,11,12,13,14,15,16,17,18,19,20,21,22,23,25,26] involved pain intensity. All comparisons underwent meta-analysis, which revealed significantly less pain in the rTMS group after treatment (SMD, −0.751, 95% CI, −0.991 to −0.511, I^2^ = 35.9%; Figure 6). The funnel plot and Egger test revealed no publication bias (*p* = 0.88).

**Figure 7:**
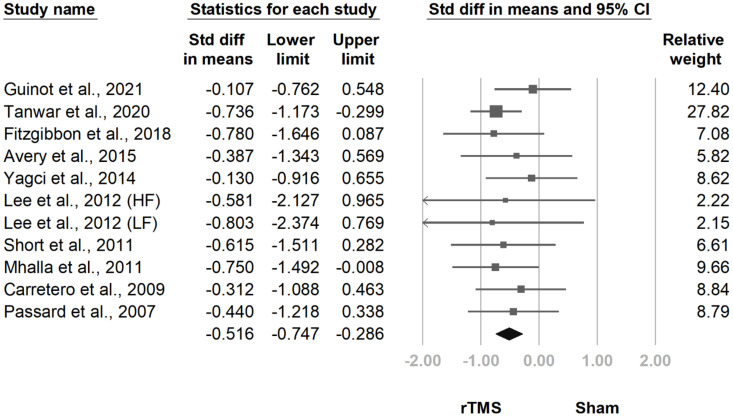
Forest plot of standardized mean differences in intensity of pain at follow-up at 2 weeks to 1 month after last treatment session. Squares indicate effect sizes of individual studies, lines indicate 95% CI, and diamond indicates the summarized effect size.

**Figure 8:**
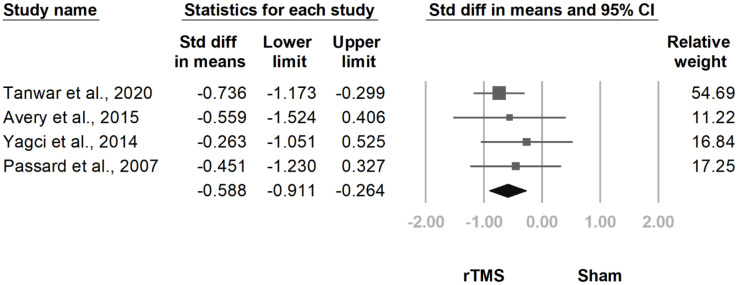
Forest plot of standardized mean differences in intensity of pain at follow-up at 1.5 to 3 months after last treatment session. Squares indicate effect sizes of individual studies, lines indicate 95% CI, and diamond indicates the summarized effect size.

At the follow-up at two weeks to one month after the final treatment session, the difference between groups remained significant (SMD, −0.516, 95% CI, −0.747 to −0.286, I^2^ = 0.0%; Figure 7). This difference persisted in the follow-up at one and a half to three months after the final treatment session (SMD, −0.588, 95% CI, −0.911 to −0.264, I^2^ = 52.6%; Figure 8). The funnel plot and Egger tests regarding the two follow-ups revealed no publication bias (*p* = 0.69; *p* = 0.21).

#### 3.3.3. BPI Interference Subscale

**Figure 9:**
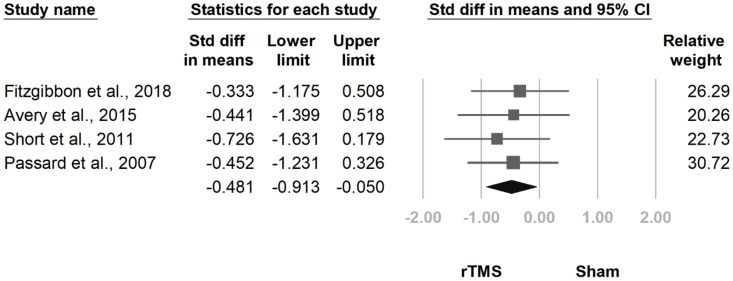
Forest plot of standardized mean differences in the interference subscale of the Brief Pain Inventory after treatment. Squares indicate effect sizes of individual studies, lines indicate 95% CI, and diamond indicates the summarized effect size.

**Figure 10:**
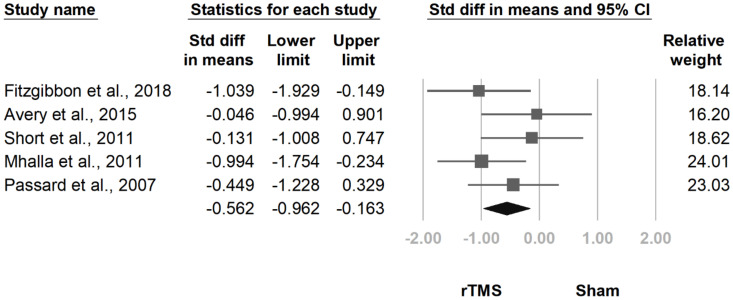
Forest plot of standardized mean differences in the interference subscale of the Brief Pain Inventory at follow-up at 2 weeks to 1 month after the last treatment session. Squares indicate effect sizes of individual studies, lines indicate 95% CI, and diamond indicates the summarized effect size.

Five articles [15,17,20,25,26] reported BPI interference subscale scores. Four [15,17,25,26] were measured immediately after the treatment and had a significant pooled effect size (SMD, −0.481, 95% CI, −0.913 to −0.050, I^2^ = 0.0%; Figure 9). At the follow-up at two weeks to one month after the final treatment session, the difference between groups remained significant (SMD, −0.562, 95% CI, −0.962 to −0.163, I^2^ = 10.8%; Figure 10). The funnel plot and Egger tests revealed no publication bias (*p* = 0.67; *p* = 0.39).

#### 3.3.4. MPQ

**Figure 11:**
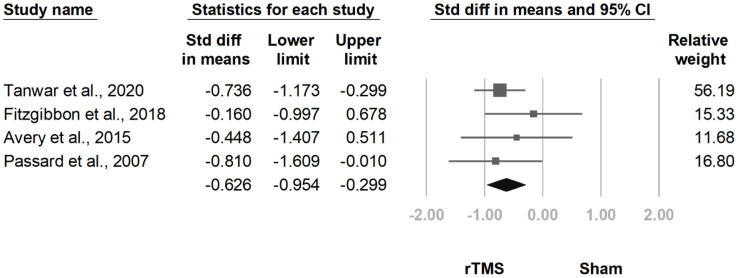
Forest plot of standardized mean differences in McGill Pain Questionnaire after treatment. Squares indicate effect sizes of individual studies, lines indicate 95% CI, and diamond indicates the summarized effect size.

**Figure 12:**
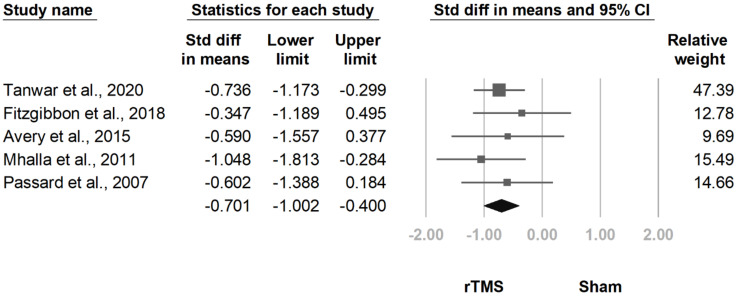
Forest plot of standardized mean differences in McGill Pain Questionnaire at follow-up at 2 weeks to 1 month after last treatment session. Squares indicate effect sizes of individual studies, lines indicate 95% CI, and diamond indicates the summarized effect size.

The MPQ was mentioned in five articles [15,17,20,23,26]. Four of the studies recorded MPQ scores after treatment, and the rTMS group exhibited a greater decrease (SMD, −0.626, 95% CI, −0.954 to −0.299, I^2^ = 0.0%; Figure 11). The effect remained significant at the follow-up at two weeks to one month after the final treatment session (SMD, −0.701, 95% CI, −1.002 to −0.400, I^2^ = 0.0%; Figure 12). The funnel plot and Egger tests did not indicate significant publication bias for either follow-up duration (*p* = 0.40; *p* = 0.63).

#### 3.3.5. Number of Tender Points

**Figure 13:**
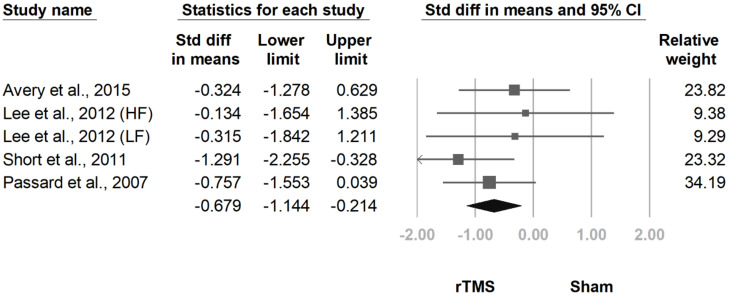
Forest plot of standardized mean differences in number of tender points after treatment. Squares indicate effect sizes of individual studies, lines indicate 95% CI, and diamond indicates the summarized effect size.

**Figure 14:**
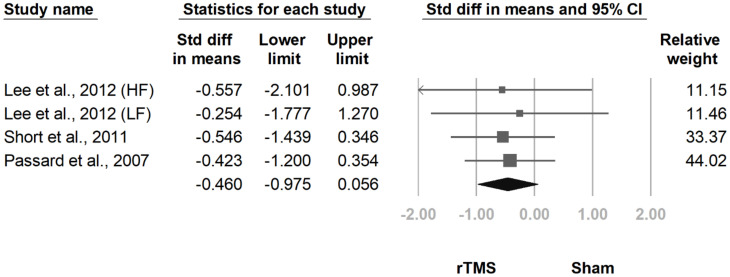
Forest plot of standardized mean differences in number of tender points at follow-up at 2 weeks to 1 month after last treatment session. Squares indicate effect sizes of individual studies, lines indicate 95% CI, and diamond indicates the summarized effect size.

Five articles [11,15,24,25,26] reported the number of tender points. Five comparisons derived from four articles [11,15,25,26] entered meta-analysis, revealing fewer tender points in the rTMS group after treatment (SMD, −0.679, 95% CI, −1.114 to −0.214, I^2^ = 0.0%; Figure 13). However, significance did not remain at the follow-up at two weeks to one month after the final treatment session (SMD, −0.460, 95% CI, −0.975 to 0.056, I^2^ = 0.0%; Figure 14). The funnel plot and Egger test revealed no publication bias in either follow-up duration (*p* = 0.39; *p* = 0.79).

#### 3.3.6. BDI

**Figure 15:**
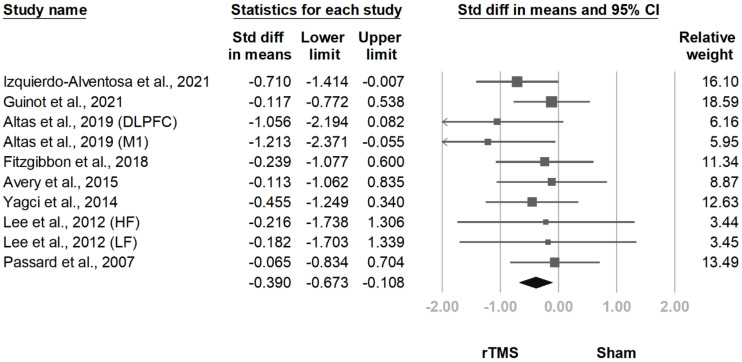
Forest plot of standardized mean differences in Beck Depression Inventory after treatment. Squares indicate effect sizes of individual studies, lines indicate 95% CI, and diamond indicates the summarized effect size.

**Figure 16:**
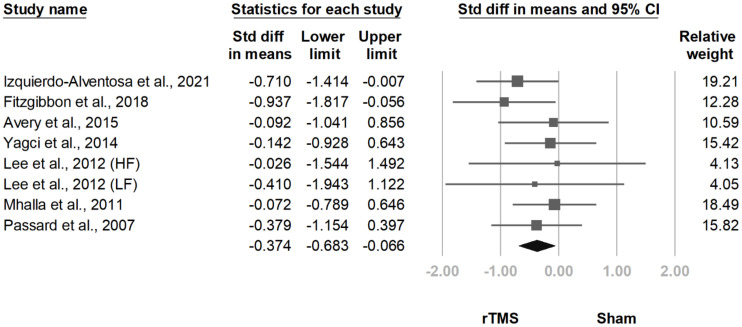
Forest plot of standardized mean differences in Beck Depression Inventory at follow-up at 2 weeks to 1 month after last treatment session. Squares indicate effect sizes of individual studies, lines indicate 95% CI, and diamond indicates the summarized effect size.

The BDI was administered in 10 trials [11,12,14,15,17,18,19,20,24,26], and 10 comparisons extracted from eight studies underwent meta-analysis. The results revealed a significantly lower BDI score in the rTMS group after treatment (SMD, −0.390, 95% CI, −0.673 to −0.108, I^2^ = 0.0%; Figure 15). The effect remained significant at the follow-up at two weeks to one month after the final treatment session (SMD, −0.374, 95% CI, −0.683 to −0.066, I^2^ = 0.0%; Figure 16). The funnel plot and Egger tests revealed no publication bias (*p* = 0.47; *p* = 0.75).

#### 3.3.7. HDRS

**Figure 17:**
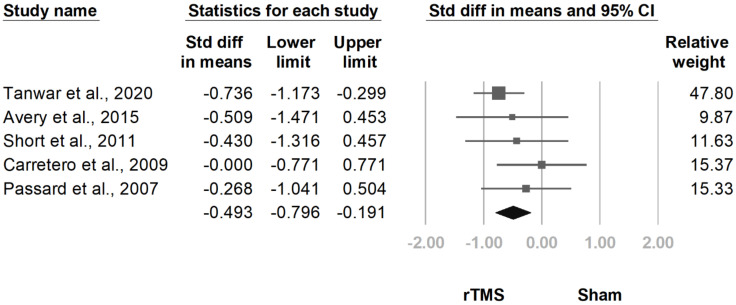
Forest plot of standardized mean differences in Hamilton Depression Rating Scale after treatment. Squares indicate effect sizes of individual studies, lines indicate 95% CI, and diamond indicates the summarized effect size.

**Figure 18:**
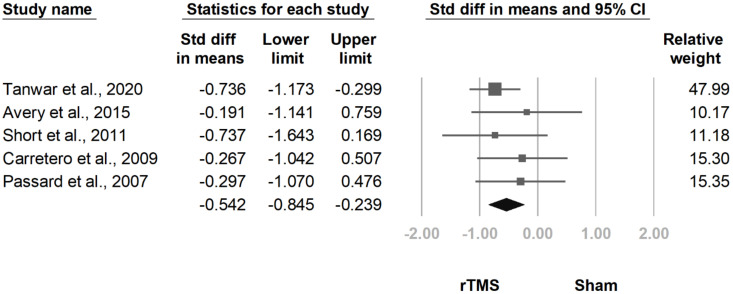
Forest plot of standardized mean differences in Hamilton Depression Rating Scale at follow-up at two weeks to one month after last treatment session. Squares indicate effect sizes of individual studies, lines indicate 95% CI, and diamond indicates the summarized effect size.

**Figure 19:**
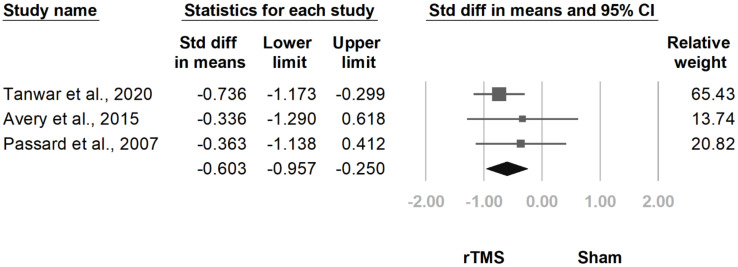
Forest plot of standardized mean differences in Hamilton Depression Rating Scale at follow-up at one and a half to three months after last treatment session. Squares indicate effect sizes of individual studies, lines indicate 95% CI, and diamond indicates the summarized effect size.

Six articles [10,15,21,23,25,26] reported HDRS scores, and five of the investigations [15,21,23,25,26] were adequate for meta-analysis. The score was lower in the rTMS group after treatment (SMD, −0.493, 95% CI, −0.796 to −0.191, I^2^ = 0.0%; Figure 17). The treatment effect persisted at the follow-ups at two weeks to one month (SMD, −0.542, 95% CI, −0.845 to −0.2339, I^2^ = 0.0%; Figure 18) and one and a half to three months (SMD, −0.603, 95% CI, −0.957 to −0.250, I^2^ = 0.0%; Figure 19) after the final treatment session. The funnel plot and Egger tests revealed no publication bias in any of these three results (*p* = 0.18; *p* = 0.18; *p* = 0.14).

#### 3.3.8. HADS-A

**Figure 20:**
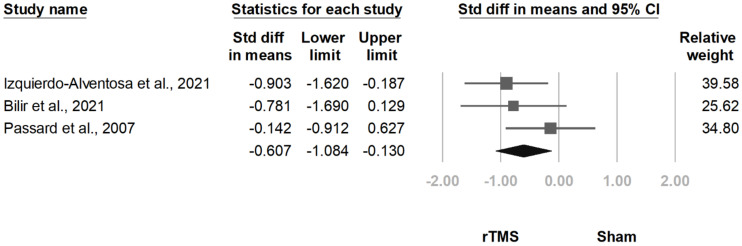
Forest plot of standardized mean differences in anxiety subscale of the Hospital Anxiety and Depression Scale after treatment. Squares indicate effect sizes of individual studies, lines indicate 95% CI, and diamond indicates the summarized effect size.

HADS-A scores were mentioned in five articles [12,13,15,20,24], and three of the studies [12,13,15] administered it immediately after the treatment. The pooled effect size results revealed a lower HADS-A score in the rTMS group (SMD, −0.607, 95% CI, −1.084 to −0.130, I^2^ = 8.9%; Figure 20). The funnel plot and Egger test indicated no publication bias (*p* = 0.99).

#### 3.3.9. FSS

**Figure 21:**
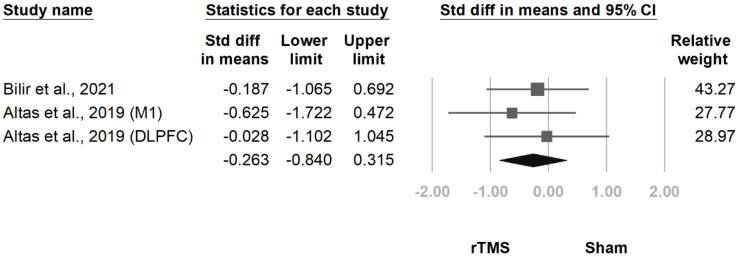
Forest plot of standardized mean differences in Fatigue Severity Scale after treatment. Squares indicate effect sizes of individual studies, lines indicate 95% CI, and diamond indicates the summarized effect size.

Three comparisons derived from two trials [13,18] measured fibromyalgia-related fatigue with the FSS. The pooled effect size was nonsignificant (SMD, −0.263, 95% CI, −0.840 to 0.315, I^2^ = 0.0%; Figure 21). The funnel plot and Egger test indicated no publication bias (*p* = 0.76).

#### 3.3.10. Subgroup Analysis

The effect sizes associated with each stimulation site is listed in Table 3. rTMS over the M1 area was effective in reducing FIQ/FIQR score, pain intensity, BPI interference subscale score, MPQ score, and BDI score; rTMS over the DLPFC reduced FIQ/FIQR score, pain intensity, MPQ score, number of tender points, and HDRS score. However, no significant difference was detected between subgroups for any outcome.

The results of the subgroups analysis by distinct diagnostic criteria are presented in Table 4. Among patients with diagnoses based on the 1990 ACR criteria, rTMS reduced FIQ/FIQR score, pain intensity, BPI interference subscale score, MPQ score, and number of tender points. For patients with diagnoses based on the 2010, 2011, or 2016 ACR criteria, rTMS reduced pain intensity, BPI interference subscale score, MPQ score, BDI score, HDRS score, and HADS-A score. Nonetheless, none of the differences between subgroups reached statistical significance.

The outcomes of the subgroup analysis by high frequency (HF) and low frequency (LF) stimulation of rTMS are shown in Table 5. For the LF group, rTMS improved FIQ/FIQR score, pain intensity, MPQ score, and HDRS score. In the HF group, FIQ/FIQR score, intensity of pain, BPI interference, MPQ score, number of tender points, BDI score, and HADS-A score improved after treatment. Nevertheless, no significant difference appeared between subgroups for any outcome.

### 3.4. Certainty of Evidence

Overall evidence was assessed using GRADE. The certainty of the evidence of the improvement of FIQ/FIQR scores after rTMS treatment revealed a low quality of evidence. The level was downgraded due to large CI and significant between-study heterogeneity. As for the outcome two weeks to one month after the last session, the certainty of the evidence was moderate. The details are presented in Table 6.

## 4. Discussion

We systematically reviewed 18 RCTs investigating the effect of rTMS on fibromyalgia-related symptoms. Compared with sham treatment, patients receiving rTMS had lower FIQ scores as well as less pain, depression, and anxiety. These effects persisted for at least two weeks after the final treatment session, and the improvement of pain and depression remained significant at up to one and a half months after the final session. Moreover, the efficacy was stronger in older patients. However, no reduction was detected in fatigue, and the correlations between FIQ score and diagnostic criteria, disease severity, and rTMS parameters were not significant.

Several systematic reviews with quantitative synthesis have yielded discrepant results. Knijnik et al. [31] performed a meta-analysis of five studies. They concluded that rTMS improved quality of life but did not reduce depression or pain. Saltychev et al. [29] conducted a meta-analysis of seven trials. They reported that the decrease of pain after rTMS did not reach clinical significance. Hou et al. [30] performed a meta-analysis of 16 studies treating fibromyalgia with NBS. Among them, 11 treated fibromyalgia with rTMS, and the pooled effect size revealed significant reductions in pain, depression, fatigue, and the number of tender points as well as general improvements in health and function. Finally, the most recent meta-analysis including 14 RCTs revealed improvements in pain intensity and FIQ score [32]. By enrolling up-to-date RCTs counting 17 in total, our meta-analysis further revealed treatment effects not only in FIQ score and pain but also in depression and anxiety. The higher statistical power in our review may explain the discrepancies. As for the effect sizes of previous meta-analyses, pain reduction was most surveyed, which had small to medium effect sizes [30,31,32,46]. This corresponds to our study, which also revealed a medium effect size [46].

The improvement in FIQ/FIQR score as well as the secondary outcomes in our study implies that an overall improvement of the total spectrum of problems related to fibromyalgia might exist, which includes fibromyalgia-related symptoms, overall impact, and functional impairment [38]. Considering the high positive correlation between FIQR and suicide risk revealed in previous articles [47,48], as well as higher health economic costs in patients with higher FIQ scores [49], this improvement in the FIQ score has an important impact at both the individual and public health levels.

We found improvements in pain intensity, pain quality, and physical functioning measured by VAS/NPRS, MPQ, and BPI interference score [50]. Although the pathophysiology of fibromyalgia is unknown, central sensitization that affects the pain modulatory system is believed to play an important role. Research has demonstrated that rTMS may attenuate symptoms of fibromyalgia by moderating the cortical excitability of brain structures associated with pain modulation [9,51]. Moreover, the M1 and DLPFC areas are crucial in top-down pain control and opioid release [23], possibly explaining the analgesic effect observed in our review. Some studies have found a relationship between the total number of tender points and the severity of central sensitization [52,53,54,55]. Hence, the decreased numbers of tender points after treatment revealed in our study may also imply an improvement in central sensitization.

Relieved emotional functioning measured by BDI, HDRS, and HADS-A were noticed by meta-analyses [50]. Similar results regarding fibromyalgia-related anxiety and depression have been reported by studies targeting the M1 [12] or DLFPC [23]. Because both anxiety and depression are related to pain [56], the symptom reduction may stem from the analgesic effects of rTMS. Furthermore, research has revealed that the DLPFC is related to the anterior insula and amygdala [57], which are associated with anxiety [58] and depressive [59] symptoms. The aforementioned evidence potentially explains the effects observed in our meta-analysis.

We discovered a positive correlation between age and FIQ score reduction in meta-regression. No studies have found this relationship between rTMS efficacy for fibromyalgia and age. However, a study compared pain sensitivity and structural changes to the brain in fibromyalgia between patients aged above and below 50 years [60]. Distinct patterns of change in the thickness of gray matter were detected, as well as increased pain sensitivity in only the older group. Moreover, insular gray matter significantly decreased with age across all patients with fibromyalgia. The authors concluded that the brain structures and functions involved in pain modulation might shift from being adaptive in younger individuals to being maladaptive in older patients with fibromyalgia. Because the insular cortex is critical for pain modulation [61] and anterior insula change is believed to be the mechanism underlying the efficacy of rTMS, we anticipate greater improvements among older patients with fibromyalgia.

We did not identify a correlation between effect size and the total number of rTMS pulses. This result may imply a ceiling of rTMS efficacy for patients with fibromyalgia. However, this result may be solely due to the insufficient power of our small sample sizes. Future studies are warranted to fill this knowledge gap.

The adverse events resulting from rTMS treatment are generally tolerable. Dizziness, nausea, headache, neck pain, stimulation site discomfort, and several neurobehavioral adverse events were reported in the enrolled studies. However, no serious adverse events were reported. Although rTMS has been reported to carry the risk of inducing seizures, no seizures were observed in our review.

The strength of our study exists in several aspects. First, we are the first to summarize fibromyalgia-related symptoms not only widely but also longitudinally. Second, this is the first meta-analysis to assess the moderators for FIQ/FIQR score, which revealed a relationship between effect size and age. This inspires future studies to assess the possible difference in pathophysiology of fibromyalgia between younger and older patients, and such correlation may also encourage succeeding randomized controlled trials to compare the effect sizes of rTMS in fibromyalgia patients of different ages. Third, we are the first to assess the moderator effect longitudinally in order to find out the possible factors that determine the duration of effectiveness. Fourth, we included up-to-date RCTs, further reducing the possibilities of false negatives compared with the previous meta-analyses [29,30,31,32].

This review and meta-analysis has several limitations. First, all of the included studies had small numbers of participants, and the patient demographics, study designs, and stimulation parameters were heterogeneous. Although, according to our meta-analyses, most potential moderators were unrelated to the treatment effects, the low statistical power meant that the possibility of false negatives was high. Second, all of the enrolled studies had a female majority. Although no correlation between treatment effect and sex was detected in meta-regression, this may result from the underrepresentation of men with fibromyalgia. Third, several distinct sets of diagnostic criteria were adopted. Only three studies used the latest 2016 ACR criteria, and the low statistical power may have caused a false negative in the detection of the correlation of criteria adopted with effect size. Moreover, the scant use of the 2016 criteria, the latest ACR criteria for fibromyalgia, might impede the generalizability of our results. Fourth, most of the included investigations allowed concurrent medication during the study period. Therefore, rTMS acted more as an add-on therapy to medication treatment. The possibility of an interaction between pharmacological and rTMS treatment effects requires further research. Fifth, we did not include participant ratings of improvement, concomitant pain treatments, deposition of participants, and adverse events as outcomes of meta-analysis because of insufficient data and high between-study heterogeneity. However, these outcomes are important in the evaluation of treatments for chronic pain [50], and future trials may estimate these outcomes to fill the gap. Sixth, FIQ/FIQR were not the primary outcome in most of the RCTs included in our review, which might bias our results. Finally, although no serious adverse events were reported, the relatively low number of participants in most of the studies may limit our ability to conclude that such events are rare [62]. Larger RCTs using the 2016 ACR criteria with a sufficient male population are warranted to confirm our findings and to delineate the optimal dose, treatment frequency, and stimulation target for rTMS.

## 5. Conclusions

This meta-analysis revealed that rTMS is safe and effective for treating multiple domains of fibromyalgia-related symptoms, and older patients may have a stronger effect. Future studies are required to detect rare adverse events and determine the optimal stimulation parameters.

## Figures and Tables

**Table 1:** Characteristics of all included studies.

Reference	Country/Region	Group	Enrolled/Completed	Diagnostic Criteria	Gender (% of Female)	Age, Years, Mean (SD)	Disease Duration, Mean (SD)
Izquierdo-Alventosa et al., 2021 [12]	Spain	PE, rTMS, control	PE: 16/16 rTMS: 17/17, control: 16/16	ACR 2016	PE: 100% rTMS: 100%, control: 100%	PE: 53.06 (8.4), rTMS: 50.47 (8.9), control: 55.13 (7.35)	NR
Guinot et al., 2021 [14]	France	rTMS, sham	rTMS: 20/17, sham: 19/19	ACR 2010	rTMS: 100%, sham: 79%	rTMS: 46.5 (10.4), sham: 42.8 (8.8)	rTMS: 11.2 years (10.9), sham: 9.2 years (9.6)
Bilir et al., 2021 [13]	Turkey	rTMS, sham	rTMS: 10/10, sham: 10/10	ACR 2016	rTMS: 100%, sham: 100%	rTMS: 46.7 (9.06), sham: 43.8 (9.37)	rTMS: median 60 months (IQR 24–63), sham: median 66 months (IQR 48.73–66)
Tanwar et al., 2020 [23]	India	rTMS, sham	rTMS: 45/45, sham: 45/41	ACR 2010	rTMS: 100%, sham: 100%	rTMS: 41.54 (8.58), sham: 39.05 (7.12)	rTMS: 8 years (5.11), sham: 7.63 years (4.65)
Cheng et al., 2019 [10]	Taiwan	rTMS, sham	rTMS: 9/9, sham: 11/10	ACR 2010	rTMS: 77.8%, sham: 70%	rTMS: median 48 (IQR 14.5), sham:51.5 (IQR 13.6)	rTMS: median 12 years (IQR 10.5), sham: median 4.5 years (IQR 17.2)
Altas et al., 2019 [18]	Turkey	M1, DLPFC, sham	M1:10/10, DLPFC: 10/10, sham: 10/10	ACR 2011	M1: 100%, DLPFC: 100%, sham: 100%	M1: 46.3 (9.01), DLPFC: 47.9 (7.89), sham: 48.2 (9.38)	M1: 3 years (1.83), DLPFC: 4.2 years (1.14), sham: 3.6 years (1.43)
Fitzgibbon et al., 2018 [17]	Australia	rTMS, sham	rTMS: 14/11, sham: 12/11	ACR 2016	rTMS: 92.9%, sham: 91.7%	rTMS: 45.07 (11.02), sham: 46.25 (15.04)	rTMS: 16 years (16.33), sham: 15.58 years (8.84)
Avery et al., 2015 [26]	USA	rTMS, sham	rTMS: 8/7, sham: 11/11	ACR 1990	rTMS: 100%, sham: 100%	rTMS: 54.86 (7.65), sham: 52.09 (10.02)	rTMS: 11 years (4.26), sham: 15.64 years (6.93)
Yagci et al., 2014 [19]	Turkey	rTMS, sham	rTMS: 14/12, sham: 14/13	ACR 1990	rTMS: 100%, sham: 100%	rTMS: 45.25 (9.33), sham: 43 (7.63)	rTMS: 53 months (29.15), sham: 54.92 months (30.44)
Tekin et al., 2014 [16]	Turkey	rTMS, sham	rTMS: 27/27, sham: 25/24	NR	rTMS: 88.9%, sham: 95.8%	rTMS: 42.4 (7.63), sham: 46.5 (8.36)	rTMS: 10.81 years (6.31), sham: 13.33 years (6.65)
Boyer et al., 2014 [24]	France	rTMS, sham	rTMS: 19/16, sham: 19/13	ACR 2010	rTMS: 100%, sham: 94.7%	rTMS: 49.1 (10.6), sham: 47.7 (10.4)	rTMS:3.7 years (4.5), sham: 3.6 years (3.8)
Maestu et al., 2013 [22]	Spain	rTMS, sham	rTMS: 28/28, sham: 28/26	ACR 1990	rTMS: 100%, sham: 100%	40.7 (6.7))	NR
Baudic et al., 2013 [27]	France	rTMS, sham	rTMS: 20/20, sham: 18/18	ACR 1990	NR	rTMS: 51.8 (11.6), sham: 49.7 (10.4)	rTMS: 13 years (12.9) sham: 11.7 years (10.2)
Lee et al., 2012 [11]	Korea	HF, LF, sham	HF rTMS: 7/5, LF rTMS: 8/5, sham: 7/5	ACR 1990	HF rTMS: 100%, LF rTMS: 100%, sham: 100%	HF rTMS: 53 (4.2), LF rTMS: 45.6 (9.6), sham: 51.3 (6.2)	HF rTMS: 57.1 months (6.4), LF rTMS: 47.2 months (20.1), sham: 44.7 months (10.3)
Short et al., 2011 [25]	USA	rTMS, sham	rTMS: 10/10, sham 10/10	ACR 1990	rTMS: 90%, sham: 78%	rTMS: 54.2 (8.28), sham: 51.67 (18.19)	rTMS: 12.1 years (7.75), sham: 10.1 years (12.81)
Mhalla et al., 2011 [20]	France	rTMS, sham	rTMS: 20/16, sham: 20/14	ACR 1990	rTMS: 100%, sham: 100%	rTMS: 51.8 (11.6), sham: 49.6 (10)	rTMS: 13 years (12.9), sham: 14.1 years (11.9)
Carretero et al., 2009 [21]	Spain	rTMS, sham	rTMS: 14/14, sham: 12/12	ACR 1990	rTMS: 100%, sham: 83.3%	rTMS: 47.5 (5.7), sham: 54.9 (4.9)	NR
Passard et al., 2007 [15]	France	rTMS, sham	rTMS: 15/13 sham: 15/13	ACR 1990	96.7%	rTMS: 52.6 (7.9), sham: 55.3 (8.9)	rTMS: 8.1 years (7.9), sham: 10.9 years (8.6)

ACR: American College of Rheumatology; DLPFC: dorsolateral prefrontal cortex; HF: high frequency; LF: low frequency; M1: primary motor cortex; NR: not reported; PE: physical exercise; rTMS: repetitive transcranial magnetic stimulation.

**Table 2:** Summary of extracted data from the included studies.

Reference	Combined Treatment	Detail of Interventions	Outcome Measure	Last Follow-Up	Adverse Event
Izquierdo-Alventosa et al., 2021 [12]	medication	Left M1, 10 Hz, 80% RMT, 3000 pulses/session in 20 min; 10 sessions/2 weeks	VAS-pain, PPT, FIQR, 6MWT, Borg CR10, 4mGST, 5STST, HADS-A, BDI, PSS, SWLS	Post-rTMS	NR
Guinot et al., 2021 [14]	multicomponent therapy program	Left M1, 10 Hz, 80% RMT, 2000 pulses/session in 20 min; 16 sessions/14 weeks	**VAS-pain**, FIQ, BDI, PSQI, PCS, PGI-C, cardiac autonomic nervous system adaptations, cardiopulmonary exercise testing	40 weeks after the first session	No adverse effects recorded
Bilir et al., 2021 [13]	medication	Left DLPFC, 10 Hz, 90% RMT, 1500 pulses/session in 15 min, 14 sessions/6 weeks	**VAS-pain**, VAS-stiffness, FIQ, FSS, HADS, ACE-R	Post-rTMS	No adverse effects recorded
Tanwar et al., 2020 [23]	medication	Right DLPFC, 1Hz, 90% RMT, 1200 pulses/session in 27 min, 20 sessions/4 weeks	**NPRS**, MPQ, HDRS, HARS, WHOQOL-BREF, NFR, pain modulation, oxidative stress markers	6 months after the last session	NR
Cheng et al., 2019 [10]	No medication allowed during the trial	Left DLPFC, 10 Hz, 100% RMT, 1600pulses/session in 20 min, 10 sessions/2 weeks	**VAS-pain**, HDRS, YMRS	Post-rTMS	Dizziness
Altas et al., 2019 [18]	medication	M1/DLPFC: left M1/DLPFC, 10 Hz, 90% RMT, 1200pulses/session in 30 min, 15 sessions/3 weeks	**VAS-pain**, **FIQ**, **FSS**, BDI, SF-36	Post-rTMS	No adverse effects recorded
Fitzgibbon et al., 2018 [17]	medication	Left DLPFC, 10 Hz, 120% RMT, 3000 pulses/session in 31.25 min, 20 sessions/4 weeks	**NPRS**, **BPI**, **MPQ**, FIQ, SF-36, ACR Fibromyalgia Scale, MFI-20, PCS, BDI, BAI, PGI-C	1 month after the last session	Site discomfort, headache, neck pain, nausea, dizziness
Avery et al., 2015 [26]	medication	Left DLPFC 10 Hz, 120% RMT, 3000 pulses/session; 15 sessions/4 weeks	NPRS, **BIRS**, BURS, MPQ, BPI, SF-36, MFI, VAS-fatigue, VAS-sleep, VAS-overall wellbeing, PPT, HDRS, BDI, cognitive tests, PGI, number of tender points	3 months after the last session	Headaches, pain at the site of stimulation, increased muscle aches, insomnia, nausea abdominal pain
Yagci et al., 2014 [19]	medication	Left M1, 1 Hz, 90% RMT, 1200 pulses/session; 10 sessions/2 weeks	VAS-pain, BDI, FIQ	3 months after the last session	Headache, tinnitus
Tekin et al., 2014 [16]	No analgesic use	Left M1, 10 Hz, 100% RMT, 1500 pulses/session; 10 sessions/2 weeks	VAS-pain, WHOQOL-BREF, MADRS	Post-rTMS	Headache
Boyer et al., 2014 [24]	medication	Left M1, 10 Hz, 90% RMT, 2000 pulses/session; 14 sessions /10 weeks	**FIQ**, SF-36, NPRS, number of tender points, PPT, BDI, HADS, FDG-PET/CT	1 week after the last session	Intercurrent medical conditions, headache
Maestu et al., 2013 [22]	Medication except acetaminophen or bromazepam were discontinued during the trial	8 Hz, 20 min; 8 sessions/8 weeks	**PPT**, blood serotonin level, VAS-daily activities, VAS-pain, VAS-fatigue, VAS-anxiety, VAS-depression, VAS-sleep, VAS-headache	Post-rTMS	No adverse effects recorded
Baudic et al., 2013 [27]	medication	Left M1, 10 Hz, 80% RMT, 1500 pulses/session, 14 sessions/21 weeks	RAVLT, SDMT, TMT, SCWT, BPI, MOS-SF-12, HADS	11 weeks after the first session	NR
Lee et al., 2012 [11]	Medication	HF rTMS: left motor cortex, 10 Hz, 80% RMT, 2000 pulses/session; 10 sessions/2 weeks; LF rTMS: right DLPFC, 1 Hz, 110% RMT, 1600 pulses/session; 10 sessions/2 weeks	Number of tender points, FIQ, VAS-pain, BDI	1 month after the last session	No adverse effects recorded
Short et al., 2011 [25]	Medication	Left DLPFC, 10 Hz, 120% RMT, 4000 pulses/session; 10 sessions/2 weeks	BPI, **NPRS**, HDRS, FIQ, number of tender points	2 weeks after the last session	Headache
Mhalla et al., 2011 [20]	Medication	Left M1, 10 Hz, 80% RMT, 1500 pulses/session, 14 sessions/21 weeks	**NPRS**, BPI, MPQ, FIQ, HADS, BDI, PCS	25 weeks after the first session	Headache, dizziness
Carretero et al., 2009 [21]	Medication	Right DLPFC, 1 Hz, 110% RMT, 1200 pulses/session in 30 min; 20 sessions/4 weeks	Likert Pain Scale, HDRS, CGI, FFS	8 weeks after the first session	Neck pain, headache, worsening of depression, nausea, tiredness
Passard et al., 2007 [15]	Medication	Left M1, 10 Hz, 80% RMT, 2000 pulses/session; 10 sessions/2 weeks	**NPRS**, BPI, MPQ, FIQ, number of tender points, PPT, HDRS, BDI, HADS	60 days after the first session	Headaches, nausea, tinnitus, dizziness

Outcomes in bold indicated being the primary outcome. ACE-R: Addenbrooke’s Cognitive Examination–last revised version; ACR: American College of Rheumatology; BAI: Beck Anxiety Inventory; BDI: Beck Depression Inventory; BIRS: Gracely Box Intensity Scale; Borg CR10: Borg Category-Ratio Scale; BPI: Brief Pain Inventory; BURS: Gracely Box Unpleasantness Rating Scales; CGI: Clinical Global Impression scale; FDG-PET/CT: fluorodeoxyglucose positron emission tomography and computed tomography; FIQ: Fibromyalgia Impact Questionnaire; FIQR: Revised Fibromyalgia Impact Questionnaire; FSS: Fatigue Severity Scale; 5STST: five-repetition sit-to-stand test; 4mGST: four-meter gait speed test; HADS: Hospital Anxiety and Depression Scale; HADS-A: anxiety subscale of HADS; HARS: Hamilton Anxiety Rating Scale; HDRS: Hamilton Depression Rating Scale; MADRS: Montgomery Asberg Rating Scale; MFI: Multidimensional Fatigue Inventory; MOS-SF-12: Medical Outcomes Study Short Form 12; MPQ: McGill Pain Questionnaire; NFR: nociceptive flexion reflex; NPRS: Numeric Pain Rating Scale; NR: not reported; PCS: pain catastrophism scale; PGI: personal global improvement; PGI-C: personal global improvement of change; PPT: pressure pain threshold; PSQI: Pittsburgh Sleep Quality Inventory; PSS: Perceived Stress Scale; RAVLT: Rey Auditory Verbal Learning Test; SCWT: Stroop Color Word Test; SDMT: Symbol Digit Modalities Test; SF-36: Short Form 36; 6MWT: six-minute walking test; SWLS: Satisfaction with Life Scale; TMT: Trail-Making Test; VAS: visual analogue scale; WHOQOL-BREF: World Health Organization Quality of Life Instrument, Short Form; YMRS: Young Mania Rating Scale.

**Table 3:** Subgroup analyses by stimulation site.

		FIQ/FIQR	Intensity of Pain	BPI-Interference	MPQ	Number of Tender Points	BDI	HDRS	HADS-A	FSS
Post-rTMS	M1	−**0.894** (−**1.707**, −**0.082**)	−**0.981** (−**1.478**, −**0.484**)	−0.452 (−1.231, 0.326)	−**0.810** (−**1.609**, −**0.010**)	−0.623 (−1.328, 0.082)	−**0.402** (−**0.739**, −**0.065**)	−0.268 (−1.041, 0.504)	−0.536 (−1.281, 0.209)	−0.028 (−1.102, 1.048)
	DLPFC	−0.426 (−0.866, 0.013)	−**0.593** (−**0.858**, −**0.328**)	−0.494 (−1.012, 0.025)	−**0.589** (−**0.949**, −**0.230**)	−**0.716** (−**1.386**, −**0.047**)	−0.364 (−0.881, 0.154)	−**0.534** (−**0.863**, −**0.205**)	−0.781 (−1.690, 0.129)	−0.358 (−1.044, 0.327)
	Total	−**0.700** (−**1.173**, −**0.228**)	−**0.751** (−**0.991**, −**0.511**)	−**0.481** (−**0.913**, −**0.050**)	−**0.626** (−**0.954**, −**0.299**)	−**0.679** (−**1.144**, −**0.214**)	−**0.390** (−**0.673**, −**0.108**)	−**0.493** (−**0.796**, −**0.191**)	−**0.607** (−**1.084**, −**0.130**)	−0.263 (−0.840, 0.315)
2 weeks to 1 month after the last session	M1	−**0.775** (−**1.363**, −**0.186**)	−0.356 (−0.713, 0.001)	−**0.728** (−**1.272**, −**0.184**)	−**0.831** (−**1.380**, −**0.283**)	−0.450 (−1.144, 0.244)	−0.318 (−0.679, 0.043)	−0.297 (−1.070, 0.476)		
	DLPFC	−**0.814** (−**1.399**, −**0.230**)	−**0.631** (−**0.933**, −**0.329**)	−0.414 (−1.039, 0.212)	−**0.645** (−**1.005**, −**0.285**)	−0.472 (−1.242, 0.299)	−0.525 (−1.120, 0.069)	−**0.586** (−**0.915**, −**0.257**)		
	Total	−**0.784** (−**1.136**, −**0.432**)	−**0.516** (−**0.747**, −**0.286**)	−**0.562** (−**0.962**, −**0.163**)	−**0.701** (−**1.002**, −**0.400**)	−0.460 (−0.975, 0.056)	−**0.374** (−**0.683**, −**0.066**)	−**0.542** (−**0.845**, −**0.239**)		
1.5 to 3 months after the last session	M1		−0.358 (−0.912, 0.196)					−0.363 (−1.138, 0.412)		
	DLPFC		−**0.706** (−**1.104**, −**0.308**)					−**0.667** (−**1.064**, −**0.269**)		
	Total		−**0.588** (−**0.911**, −**0.264**)					−**0.603** (−**0.957**, −**0.250**)		

All values are stated as standardized mean differences (95% CI). Bold values indicate significant differences between groups. BDI: Beck Depression Inventory; BPI-interference: interference subscale of Brief Pain Inventory; DLPFC: dorsolateral prefrontal cortex; FSS: Fatigue Severity Scale; FIQ: Fibromyalgia Impact Questionnaire; FIQR: Revised Fibromyalgia Impact Questionnaire; HADS-A: anxiety subscale of Hospital Anxiety and Depression Scale; HDRS: Hamilton Depression Rating Scale; M1: primary motor cortex; MPQ: McGill Pain Questionnaire.

**Table 4:** Subgroup analyses by diagnosis criteria.

		FIQ/FIQR	Intensity of Pain	BPI-Interference	MPQ	Number of Tender Points	BDI	HDRS	HADS-A	FSS
Post-rTMS	ACR 1990	−**1.015** (−**1.474**, −**0.557**)	−**0.640** (−**0.917**, −**0.363**)	−**0.534** (−**1.036**, −**0.031**)	−**0.661** (−**1.275**, −**0.047**)	−**0.679** (−**1.144**, −**0.214**)	−0.215 (−0.651, 0.222)	−0.271 (−0.689, 0.148)	−0.142 (−0.912, 0.627)	--^a^
	ACR 2010/2011/2016	−0.552 (−1.245, 0.200)	−**0.723** (−**1.163**, −**0.283**)	−0.333 (−1.175, 0.508)	−**0.563** (−**1.081**, −**0.045**)	--^a^	−**0.528** (−**0.920**, −**0.135**)	−**0.736** (−**1.173**, −**0.299**)	−**0.856** (−**1.419**, −**0.293**)	−0.263 (−0.840, 0.315)
	Total	−**0.700** (−**1.173**, −**0.228**)	−**0.751** (−**0.991**, −**0.511**)	−**0.481** (−**0.913**, −**0.050**)	−**0.626** (−**0.954**, −**0.299**)	−**0.679** (−**1.144**, −**0.214**)	−**0.390** (−**0.673**, −**0.108**)	−**0.493** (−**0.796**, −**0.191**)	−**0.607** (−**1.084**, −**0.130**)	−0.263 (−0.840, 0.315)
2 weeks to 1 month after the last session	ACR 1990	−**0.776** (−**1.160**, −**0.391**)	−**0.461** (−**0.779**, −**0.144**)	−**0.460** (−**0.887**, −**0.033**)	−**0.773** (−**1.250**, −**0.296**)	−0.460 (−0.975, 0.056)	−0.179 (−0.552, 0.194)	−0.362 (−0.782, 0.058)		
	ACR 2010/2011/2016	−0.827 (−1.698, 0.043)	−**0.557** (−**0.968**, −**0.146**)	−**1.039** (−**1.929**, −**0.149**)	−**0.654** (−**1.042**, −**0.266**)	--^a^	−**0.799** (−**1.348**, −**0.249**)	−**0.736** (−**1.173**, −**0.299**)		
	Total	−**0.784** (−**1.136**, −**0.432**)	−**0.516** (−**0.747**, −**0.286**)	−**0.562** (−**0.962**, −**0.163**)	−**0.701** (−**1.002**, −**0.400**)	−0.460 (−0.975, 0.056)	−**0.374** (−**0.683**, −**0.066**)	−**0.542** (−**0.845**, −**0.239**)		
1.5 to 3 months after the last session	ACR 1990		−0.408 (−0.888, 0.072)					−0.352 (−0.954, 0.249)		
	ACR 2010/2011/2016		−**0.736** (−**1.173**, −**0.299**)					−**0.736** (−**1.173**, −**0.299**)		
	Total		−**0.588** (−**0.911**, −**0.264**)					−**0.603** (−**0.957**, −**0.250**)		

All values are stated as standardized mean differences (95% CI). Bold values indicate significant differences between groups. ^a^ No studies are available to calculate the effect size. ACR: American College of Rheumatology; BDI: Beck Depression Inventory; BPI-interference: interference subscale of Brief Pain Inventory; FSS: Fatigue Severity Scale; FIQ: Fibromyalgia Impact Questionnaire; FIQR: Revised Fibromyalgia Impact Questionnaire; HADS-A: anxiety subscale of Hospital Anxiety and Depression Scale; HDRS: Hamilton Depression Rating Scale; MPQ: McGill Pain Questionnaire; rTMS: repetitive transcranial.

**Table 5:** Subgroup analyses by stimulation frequency.

		FIQ/FIQR	Intensity of Pain	BPI-Interference	MPQ	Number of Tender Points	BDI	HDRS	HADS-A	FSS
Post-rTMS	LF	−**0.906** (−**1.637**, −**0.174**)	−**0.652** (−**0.989**, −**0.316**)	--^a^	−**0.736** (−**1.173**, −**0.299**)	−0.315, (−1.842, 1.211)	−0.396 (−1.100, 0.308)	−0.439 (−1.147, 0.268)	--^a^	--^a^
	HF	−**0.664** (−**1.227**, −**0.100**)	−**0.808** (−**1.162**, −**0.453**)	−**0.481** (−**0.913**, −**0.050**)	−0.4863 (−0.981, 0.010)	−**0.716** (−**1.205**, −**0.228**)	−**0.389** (−**0.697**, −**0.081**)	−0.384 (−0.882, 0.114)	−**0.607** (−**1.084**, −**0.130**)	−0.263 (−0.840, 0.315)
	Total	−**0.700** (−**1.173**, −**0.228**)	−**0.751** (−**0.991**, −**0.511**)	−**0.481** (−**0.913**, −**0.050**)	−**0.626** (−**0.954**, −**0.299**)	−**0.679** (−**1.144**, −**0.214**)	−**0.390** (−**0.673**, −**0.108**)	−**0.493** (−**0.796**, −**0.191**)	−**0.607** (−**1.084**, −**0.130**)	−0.263 (−0.840, 0.315)
2 weeks to 1 month after the last session	LF	−0.670 (−1.386, 0.046)	−**0.550** (−**0.885**, −**0.215**)	--^a^	−**0.736** (−**1.173**, −**0.299**)	−0.254 (−1.777, 1.270)	−0.198 (−0.897, 0.501)	−**0.615** (−**1.018**, −**0.213**)		
	HF	−**0.824** (−**1.272**, −**0.377**)	−**0.486** (−**0.804**, −**0.168**)	−**0.562** (−**0.962**, −**0.163**)	−**0.669** (−**1.084**, −**0.255**)	−0.486 (−1.024, 0.062)	−**0.417** (−**0.761**, −**0.073**)	−0.402 (−0.902, 0.098)		
	Total	−**0.784** (−**1.136**, −**0.432**)	−**0.516** (−**0.747**, −**0.286**)	−**0.562** (−**0.962**, −**0.163**)	−**0.701** (−**1.002**, −**0.400**)	−0.460 (−0.975, 0.056)	−**0.374** (−**0.683**, −**0.066**)	−**0.542** (−**0.845**, −**0.239**)		
1.5 to 3 months after the last session	LF		−**0.618**, (−**1.020**, −**0.216**)					−**0.736** (−**1.173**, −**0.299**)		
	HF		−0.494 (−1.100, 0.112)					−0.352 (−0.954, 0.249)		
	Total		−**0.588** (−**0.911**, −**0.264**)					−**0.603** (−**0.957**, −**0.250**)		

All values are stated as standardized mean differences (95% CI). Bold values indicate significant differences between groups. ^a^ No studies are available to calculate the effect size. BDI: Beck Depression Inventory; BPI-interference: interference subscale of Brief Pain Inventory; FSS: Fatigue Severity Scale; FIQ: Fibromyalgia Impact Questionnaire; FIQR: Revised Fibromyalgia Impact Questionnaire; HADS-A: anxiety subscale of Hospital Anxiety and Depression Scale; HDRS: Hamilton Depression Rating Scale; HF: high frequency; LF: low frequency; MPQ: McGill Pain Questionnaire; rTMS: repetitive transcranial magnetic stimulation.

**Table 6:** Certainty of evidence for improvement of FIQ/FIQR scores after treatment.

Quality Assessment						Summary of Findings, SMD (95% CI)		
Number of Participants (Studies), Follow-Up Period	Risk of Bias	Inconsistency	Indirectness	Imprecision	Publication Bias	Sham	rTMS	Certainty of Evidence
228 (9), immediately post-intervention	No serious limitation ^a^	Serious limitation ^b^	No serious limitation ^c^	Serious limitation ^d^	Undetectable	−0.383 (−0.597, −0.170) ^e^	−1.165 (−1.492, −0.837) ^f^	Low ⨁⨁◯◯
139 (6), 2 weeks to 1 month after the last session	No serious limitation ^a^	No serious limitation ^b^	No serious limitation ^c^	Serious limitation ^d^	Undetectable	−0.387 (−0.719, −0.055) ^g^	−1.157 (−1.579, −0.735) ^h^	Moderate ⨁⨁⨁◯

CI: confidence interval; FIQ: Fibromyalgia Impact Questionnaire; FIQR: Revised Fibromyalgia Impact Questionnaire; rTMS: repetitive transcranial magnetic stimulation; SMD: standardized mean difference. ^a^ Most studies included scored low risk of bias during assessment. ^b^ The I^2^ was over 50% during the first follow-up and below 50% at the second follow-up. ^c^ No indirectness was detected in this outcome. ^d^ The upper and lower limit of 95% CI ranged from large to small effect size. ^e^ This was calculated by pooling the sham group of the 11 comparisons included in the primary outcome, comparing the FIQ/FIQR score before and after treatment. ^f^ This was calculated by pooling the rTMS group of the 11 comparisons included in the primary outcome, comparing the FIQ/FIQR score before and after treatment. ^g^ This was calculated by pooling the rTMS group of the 5 studies included in the primary outcome and provided baseline data, comparing the FIQ/FIQR score before and 2 weeks to 1 month after treatment. ^h^ This was calculated by pooling the rTMS group of the 5 studies included in the primary outcome and provided baseline data, comparing the FIQ/FIQR score before and 2 weeks to 1 month after treatment.

## Data Availability

No new data were created in this study.

## Supplementary Materials

The following are available online at www.mdpi.com/article/10.3390/jcm10204669/s1, File S1: PRISMA checklist and search strategies for systematic review and meta-analysis, File S2: Bubble plot for meta-regression.
