# Supplementary material for: Efficacy of Repetitive Transcranial Magnetic Stimulation in Fibromyalgia: A Systematic Review and Meta-Analysis of Randomized Controlled Trials
Source: J Clin Med. 2021 Oct 12;10(20):4669. doi: 10.3390/jcm10204669 (PMC8538417; doi:10.3390/jcm10204669)
Supplement: Supplementary file 1 [file jcm-10-04669-s001.zip › File S2.pdf]

File S2. Bubble plots for meta-regression

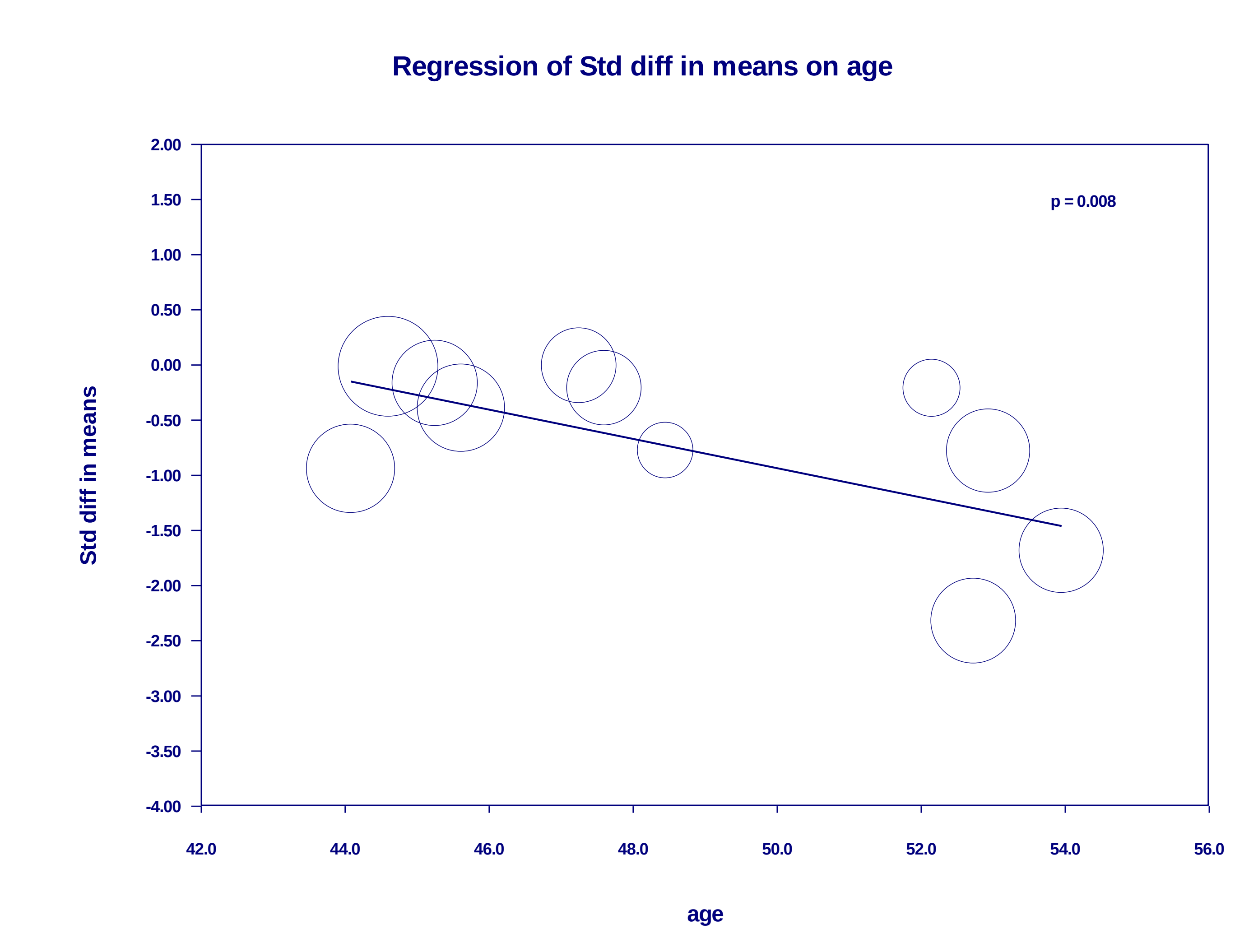

(a)[a]

(a) Bubble plot for meta-regression between age and effect sizes.

[a]Size of the circles indicated the weight of each included study.
